# Assessment of axillary node status by ultrasound after neoadjuvant chemotherapy in patients with clinically node-positive breast cancer according to breast cancer subtype

**DOI:** 10.1038/s41598-021-89738-8

**Published:** 2021-05-25

**Authors:** Yurina Maeshima, Takehiko Sakai, Akiko Ogiya, Yoko Takahashi, Yumi Miyagi, Yumi Kokubu, Tomo Osako, Yoshinori Ito, Shunji Takahashi, Shinji Ohno, Takayuki Ueno

**Affiliations:** 1 Department of Surgical Oncology, Breast Oncology Center, Cancer Institute Hospital of Japanese Foundation for Cancer Research, 3-8-31, Ariake, Koto-ku, Tokyo, 135-8550 Japan; 2 Department of Ultrasound, Cancer Institute Hospital of Japanese Foundation for Cancer Research, Tokyo, Japan; 3 Division of Pathology, Cancer Institute of Japanese Foundation for Cancer Research, Tokyo, Japan; 4 Department of Breast Medical Oncology, Cancer Institute Hospital of Japanese Foundation for Cancer Research, Tokyo, Japan; 5 Medical Oncology, Cancer Institute Hospital of the Japanese Foundation for Cancer Research, Tokyo, Japan

**Keywords:** Medical research, Oncology

## Abstract

The use of sentinel node biopsy (SNB) following neoadjuvant chemotherapy (NAC) for patients with cN1 breast cancer is controversial. Improvements of negative predictive value (NPV) by axillary ultrasound (AUS), which corresponds to the accurate prediction rate of node-negative status after NAC, would lead to decreased FNR of SNB following NAC. In this study, we retrospectively investigated the accurate prediction rate of NPV by AUS after NAC in patients with cytologically node-positive breast cancer treated between January 2012 and December 2016. Of 279 eligible patients, the NPV was 49.2% in all patients, but varied significantly by tumor subtype (*p* < 0.001) and tumor response determined by magnetic resonance imaging (MRI) (*p* = 0.0003). Of the 23 patients with clinically node negative (ycN0) by AUS and clinical complete response in primary lesion by MRI, the NPV was 100% in patients with HR±/HER2+ or HR−/HER2− breast cancer. In conclusion, regarding FNR reduction post-NAC, it will be of clinical value to take tumor subtype and primary tumor response using MRI into account to identify patients for SNB after NAC.

## Introduction

Neoadjuvant chemotherapy (NAC) is widely used as a perioperative systemic therapy for operable breast cancer to reduce the tumor volume and nodal stage, to evaluate chemosensitivity and to facilitate translational research^1^. A pathological complete response (pCR) occurs in 40% to 50% of patients with human epidermal growth factor receptor-2 (HER2)-positive and triple-negative breast cancer^2^. Although axillary dissection is currently considered standard for patients with clinically node-positive breast cancer, a more conservative approach may be applicable if patients achieve pCR in the axilla after NAC. However, the use of sentinel lymph node biopsy (SNB) following NAC for patients with cN1 breast cancer is controversial even if nodal status becomes clinically negative after NAC. Following NAC, lymphatic drainage from the breast can be impaired and tumor regression in the axilla can take a nonuniform pattern, leading to an increased false-negative rate (FNR)^3^. Three major prospective trials previously evaluated the use of SNB in patients with clinically node-positive breast cancer receiving NAC: American College of Surgeons Oncology Group (ACOSOG) Z1071 trial, SENTINA trial, and SN FNAC trial. These three trials reported respective FNRs of 12.6%, 14.2%, and 13.4%, all of which were above the currently accepted cut off of 10%^3–5^. In the ACOSOG Z1071 trial, patients underwent SNB regardless of nodal response to NAC, but when patients with normal ultrasound (US) findings in the axilla after NAC were selected, FNR was reduced from 12.6 to 9.8%^6^. However, although 70.4% of patients had normal lymph node-US findings after NAC, nodal pCR was only 39.0%, which suggests that normal-appearing lymph nodes on US does not preclude residual disease within lymph nodes on final pathology^6^. Although preoperative axillary imaging assessment may help to decide axillary surgery procedure after NAC, AUS is not commonly used to assess axillary response to NAC.

It is conceivable that improvements of negative predictive value (NPV) by AUS, which corresponds to the accurate prediction rate of node-negative status after NAC, would lead to decreased FNR of SNB following NAC. Therefore, in this study, we retrospectively investigated the accurate prediction rate of NPV by AUS after NAC in patients with cytologically node-positive breast cancer and investigated factors related to the rate.

## Materials and methods

### Patients

From January 2012 through December 2016, 298 patients with clinical stage T1–T4, N1–N2, M0 primary breast cancer with cytologically proven axillary metastasis, who underwent surgery including axillary lymph node dissection (ALND) following NAC, were retrospectively reviewed. All the patients received fine needle aspiration in axillary lymph node before NAC, which proved cytologically positive axillary node metastasis. Patients received anthracycline- and/or taxane-based regimens. Patients with HER2-positive breast cancer received anti-HER2 therapy. HER2 amplification status was determined by fluorescence in situ hybridization (FISH) and/or positive immunohistochemistry findings. Patient exclusion criteria included no nodal assessment by AUS at our institution and two or fewer cycles of chemotherapy for NAC.

The protocol has been approved by the institutional ethical committee (Cancer Institute Hospital of Japanese Foundation for Cancer Research) (No.2018-1100). The present study was carried out in accordance with the relevant guidelines and regulations/ethical principles of the Declaration of Helsinki.

### Imaging diagnosis

Clinical staging was determined by mammogram, breast ultrasonography, AUS, abdominal ultrasonography, magnetic resonance imaging (MRI) and bone scintigraphy, or positron emission tomography-computed tomography in all patients with node-positive disease. We evaluated NAC efficacy by breast ultrasonography, AUS, and contrast-enhanced MRI before NAC, at changes in chemotherapy regimen, and after NAC.

If there were any concerns regarding disease progression, imaging studies were conducted. Tumor response assessment by MRI was performed by measuring the maximum tumor diameter before and after NAC based on Response Evaluation Criteria in Solid Tumors (RECIST) version 1.1.

### AUS and MRI imaging

**Figure 1:**
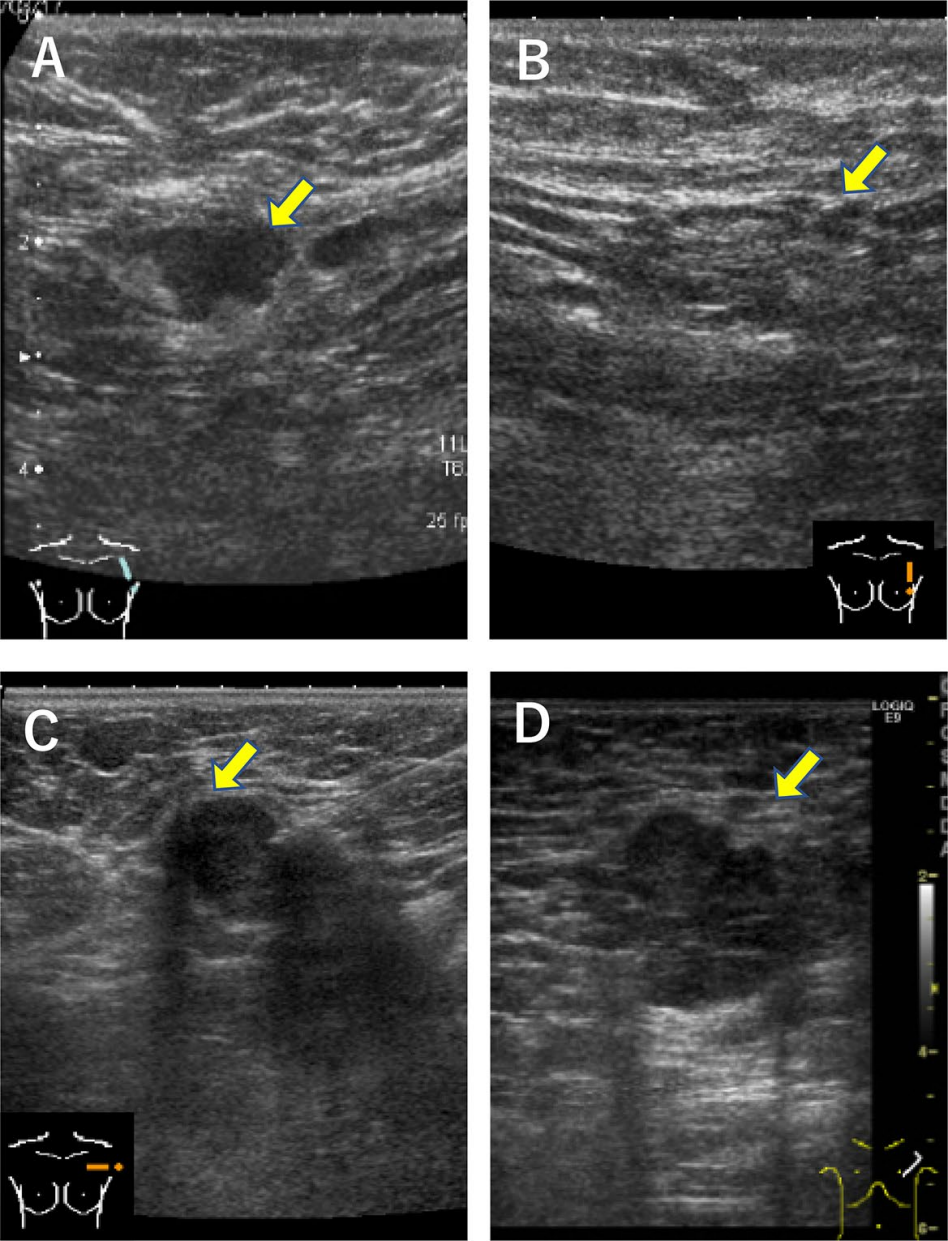
Representative ultrasound images. Patient 1. Before NAC, oval shaped lymph node was detected, which was cytologically confirmed as metastatic node (**A**). After NAC, lymph node shrunk, which was pathologically confirmed as negative node (**B**). Patient 2. Before NAC, ultrasound image of an axillary lymph node on fine needle aspiration showed irregular shaped lymph node, which was cytologically confirmed as metastatic node (**C**). After NAC, irregular shaped lymph node remained, which was pathologically confirmed as positive node (**D**).

AUS was conducted by an experienced breast sonographer. A clinically positive lymph node was defined as having a concentric cortical thickness > 3 mm, absent fatty hilum, or irregular morphology (Fig. 1). To standardize evaluation of nodal status after NAC, lymph node status was assessed by both breast radiologists and breast surgeons. All breast MRI images were evaluated by two experienced radiologists. Tumor extent, morphology, and relative enhancement were assessed during initial and late enhancements at baseline and during NAC using MRI images. The extent of each tumor was assessed by its largest diameter in three reformatted planes (sagittal, axial, and coronal) at initial and late enhancements. Radiological complete response in breast MRI was defined as the absence of dynamic contrast-enhancement on T1-weighted MRI series.

### Axillary surgery

All patients received ALND regardless of clinical response. A dissection of Levels I and II was conducted for all patients. For suspected Level II lymph nodes identified by palpation during surgery, intraoperative frozen section analysis was performed. In cases of nodes positive for Level II, a Level III dissection was performed.

### Pathological evaluation

Estrogen receptor (ER), progesterone receptor (PgR), and HER2 status were determined on core biopsy samples obtained prior to NAC. ER- and PgR-positive status (hormone receptor positive, HR +) were defined as ≥ 10% ER- and PgR-positive cancer cells by immunohistochemistry, and HER2 positive was defined either by an immunohistochemical HER2 score of 3+ or a score of 2+ with evidence of gene amplification by FISH. Lymph nodes were cut in half, and interior surfaces were stained using hematoxylin and eosin. Axillary pCR was defined as no evidence of metastatic carcinoma, including absence of isolated tumor cell clusters in lymph node.

### Statistical analysis

Chi-square test was used to compare tumor characteristics between pathological and AUS nodal assessments, and *p*-values < 0.05 were considered statistically significant. All analyses were performed using JMP Pro 15 (SAS Institute Inc., Cary, NC, USA).

### Ethical approval

The protocol has been approved by the institutional ethical committee (Cancer Institute Hospital of Japanese Foundation for Cancer Research) (No. 2018-1100). Due to the retrospective nature of this study the need for informed consent was waived.

## Results

### Patient characteristics

**Figure 2:**
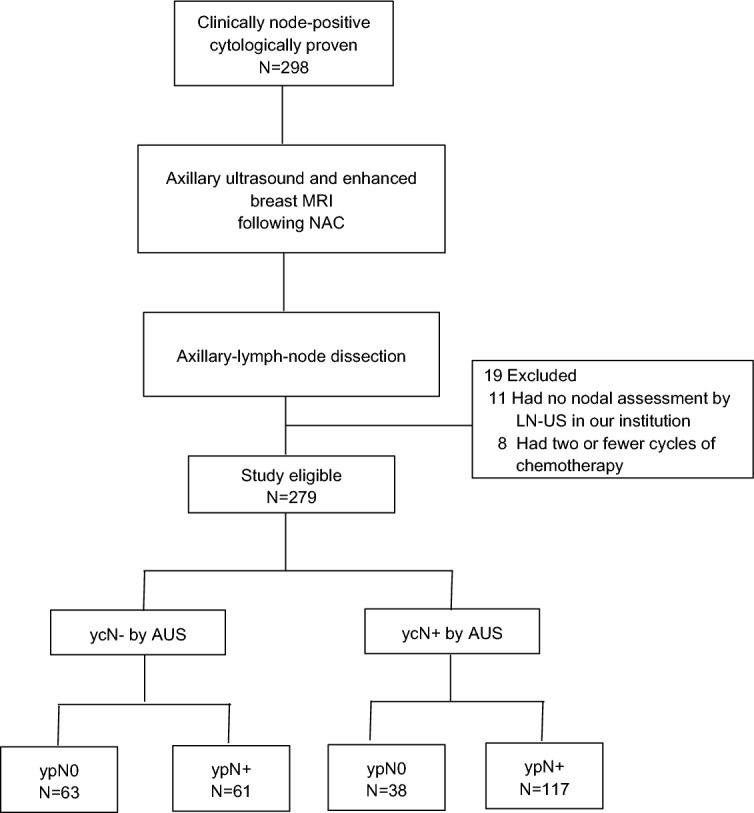
Flow diagram of the study. AUS axillary ultrasound, NAC neoadjuvant chemotherapy.

Our initial cohort consisted of 298 patients with clinical stage T1–T4, N1–N2, M0 primary breast cancer and cytologically proven axillary metastasis who underwent surgery with ALND following NAC. Eligibility criteria were met by 279 patients, and 19 patients were excluded because 11 had no nodal assessment by AUS at our institution and 8 had two or fewer cycles of chemotherapy (Fig. 2).

Patient characteristics and treatment are presented in Table 1 by pathological and clinical nodal status after NAC. The majority of chemotherapy regimens included both anthracycline and taxane (96.8%), and all patients with HER2+ disease received an anti-HER2+ regimen. Clinical tumor responses in the breast assessed by MRI were complete response (CR) in 38 patients (13.6%), partial response (PR) in 193 (69.2%), stable disease (SD) in 40 (14.3%), and progressive disease (PD) in 8 (2.9%). The median number of nodes removed at surgery was 16 (range 8–37). The median number of pathologically positive residual nodes was 2 (range 1–16) in the whole cohort of patients with ypN+ . Of the 61 patients with ycN0ypN+ , 26 (42.6%) had 1 positive lymph node on pathological review, 9 (14.8%) had 2 positive lymph nodes, 7 (11.5%) had 3 positive lymph nodes, and 19 (31.1%) had more than 3 positive lymph nodes (Table 1).

**Table 1:** Patient characteristics by pathological and clinical nodal status after NAC.

Characteristics	No. of patients (%)				
	ycN0 (n = 124)		ycN+ (n = 155)		All (n = 279)
	ycN0ypN0 (n = 63)	ycN0ypN+ (n = 61)	ycN+ ypN0 (n = 38)	ycN+ ypN+ (n = 117)	
**Clinical T category at diagnosis**					
T1	7 (11.1)	6 (9.8)	7 (18.4)	16 (13.7)	36 (12.9)
T2	50 (79.3)	42 (68.9)	25 (65.8)	79 (67.5)	196 (70.3)
T3	4 (6.3)	7 (11.5)	2 (5.3)	12 (10.3)	25 (9.0)
T4	2 (3.2)	6 (9.8)	4 (10.5)	10 (8.5)	22 (7.9)
**Approximated subtype**					
HR+/HER2−	22 (34.9)	52 (85.2)	15 (39.5)	94 (80.3)	183 (65.6)
HR+/HER2+	17 (27.0)	2 (3.3)	6 (15.8)	7 (6.0)	32 (11.5)
HR−/HER2+	13 (20.6)	2 (3.3)	7 (18.4)	2 (1.7)	24 (8.6)
HR−/HER2−	11 (17.5)	5 (8.2)	10 (26.3)	14 (12.0)	40 (14.3)
**Tumor histology**					
IDC	57 (90.5)	52 (85.2)	16 (42.1)	103 (88.0)	228 (81.7)
ILC	2 (3.2)	1 (1.6)	0	1 (0.9)	4 (1.4)
Others	4 (6.3)	8 (13.1)	22 (57.9)	13 (11.1)	47 (16.8)
**Neoadjuvant chemotherapy regimen**					
Anthracyclin and a taxane*	61 (96.8)	61 (100)	35 (92.1)	113 (96.6)	270 (96.8)
Anthracyclin-based	1 (1.6)	0	0	3 (2.6)	4 (1.4)
Taxane-based	0 (0)	0	3 (7.9)	1 (0.9)	4 (1.4)
Anti-HER2 regimen**	30 (47.6)	4 (6.6)	13 (34.2)	9 (7.7)	56 (20.1)
**No. of positive nodes before NAC as assessed by AUS**					
1	20 (31.7)	22 (36.0)	9 (23.7)	29 (24.8)	80 (28.7)
2	17 (27.0)	11 (18.0)	10 (26.3)	21 (17.9)	59 (21.1)
3	7 (1.1)	8 (13.1)	4 (10.5)	20 (17.0)	39 (14.0)
≧4	19 (30.2)	20 (32.8)	15 (2.6)	47 (40.1)	101 (36.2)
**No. of positive nodes after NAC as assessed by AUS**					
0	63	61	0	0	123 (44.1)
1	0	0	23 (60.5)	61 (52.1)	84 (30.1)
2	0	0	6 (15.8)	25 (21.4)	31 (39.2)
3	0	0	6 (15.8)	11 (9.4)	17 (6.1)
≧4	0	0	3 (7.9)	20 (17.0)	23 (8.2)
**No. of nodes removes [median (range)]**	16 (8–29)	16 (9–32)	16.5 (9–33)	16 (9–37)	16 (8–37)
**No. of positive residual nodes at ALND after NAC**					
0	63	0	38	0	101 (36.2)
1	0	26 (42.6)	0	48 (41.0)	74 (26.5)
2	0	9 (14.8)	0	22 (18.8)	31 (11.1)
3	0	7 (11.5)	0	16 (13.7)	23 (8.2)
≧4	0	19 (31.1)	0	31 (26.5)	50 (17.9)
**Tumor response as assessed by MRI**					
CR	20 (31.7)	3 (4.9)	14 (36.8)	1 (0.9)	38 (13.6)
PR	42 (66.7)	50 (82.0)	20 (52.6)	81 (69.2)	193 (69.2)
SD	1 (1.6)	7 (11.5)	2 (5.3)	30 (25.6)	40 (14.3)
PD	0	1 (1.6)	2 (5.3)	5 (4.3)	8 (2.9)
**Type of surgery**					
Total mastectomy	42 (66.7)	18 (29.5)	14 (36.8)	88 (75.2)	162 (58.0)
Partial mastectomy	21 (33.3)	43 (70.5)	24 (63.2)	29 (24.8)	117 (41.9)

*ER* estrogen receptor, *HER2* human epidermal growth factor receptor 2, *IDC* invasive ductal carcinoma, *ILC* invasive lobular carcinoma, *AUS* axillary ultrasound, *NAC* neoadjuvant chemotherapy, *ALND* axillary lymph node dissection.

*Two patients participated in clinical trial JBCRG17 and received an anthracycline- and taxane-based regimen plus eribulin.

**Two patients participated in the NeoPeak clinical trial and received either pertuzumab and T-DM1 or pertuzumab, trastuzumab, docetaxel, and carboplatin.

### Axillary pCR rate by tumor subtype

**Figure 3:**
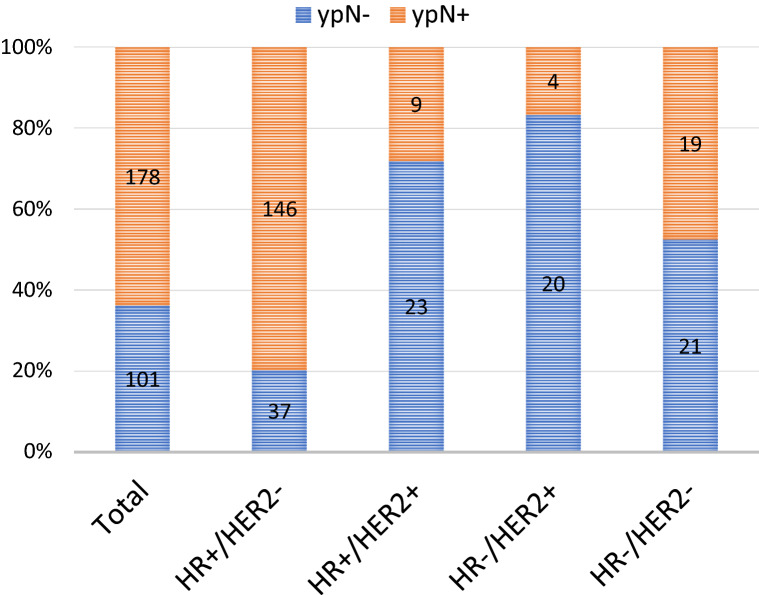
Pathological nodal status at surgery according to tumor subtype. *HR* hormone receptor, *HER2* human epidermal growth factor receptor 2.

Of our 279 patients, 101 patients (36.2%) were pathologically confirmed axillary node-negative (ypN0). The rate of ypN0 was 20.2% (37/183) in HR+ /HER2−, 71.9% (23/32) in HR+ /HER2+ , 83.3% (20/24) in HR−/HER2+ , and 52.5% (21/40) in HR−/HER2− patients (Fig. 3).

### Sensitivity and specificity of AUS

Of our 279 patients, 124 patients (44.4%) were clinically node negative by AUS after NAC (ycN0), of whom 63 patients (50.8%) were pathologically node negative at surgery (ypN0). Of 155 patients with clinically node positive by AUS after NAC (ycN +), only 38 patients (24.5%) had nodal pCR (Table 1, Fig. 2). The sensitivity and specificity of AUS after NAC were 65.7% and 62.3%, respectively (Table 2). We also determined that the negative predictive value (NPV), defined as the accurate prediction rate of node-negative status after NAC by AUS, was 50.8% in our total cohort and 29.7% in HR+ /HER2−, 89.5% in HR+ /HER2+ , 86.7% in HR−/HER2+ , and 68.8% in HR−/HER2− patients (Table 3). NPV was highest in patients with HER2+ breast cancer and lowest in patients with HR+ /HER2− breast cancer. The accuracy of AUS, which is calculated as (true positive; TP+ true negative; TN)/(true positive; TP+ true negative; TN+ false positive; FP+ false negative; FN), is 63.4% in HR+ /HER2−, 75% in HR+ /HER2+ , 62.5% in HR−/HER2+ , and 62.5% in HR−/HER2−.

**Table 2:** Clinical and pathological nodal assessment after NAC.

	ycN+	ycN−	
ypN+	117	61	Sensitivity = 65.7%
ypN−	38	63	Specificity = 62.3%
	Positive predictive value (PPV) = 75.5%	Negative predictive value (NPV) = 50.8%	

*AUS* axillary lymph node ultrasound.

**Table 3:** Accurate prediction rate of nodal status after NAC determined by AUS according to tumor subtype.

Tumor subtype	PPV (%)	NPV (%)
HR+/HER2−	86.2	29.7
HR+/HER2+	53.8	89.5
HR−/HER2+	22.2	86.7
HR−/HER2−	58.3	68.8

*NAC* neoadjuvant chemotherapy, *AUS* axillary lymph node ultrasound, *HR* hormone receptor*, HER2* human epidermal growth factor receptor 2.

### Factors related to NPV

Comparison of tumor characteristics between patients with ycN0ypN0 and patients with ycN0ypN+ are presented in Table 4. We found that the NPV by AUS varied significantly by tumor subtype (*p* < 0.001) and tumor response as assessed by MRI after NAC completion (*p* < 0.0003); however, there was no significant difference between the two groups regarding tumor category at diagnosis, tumor histology, and the number of positive nodes before NAC as assessed by AUS. Of the 23 patients who achieved ycN0 by AUS and breast cCR by MRI, we found that the accurate prediction rate of ypN0 was 100% in patients with HR±/HER2+ or HR−/HER2− breast cancer, and only 57% in HR+ /HER2− breast cancer.

**Table 4:** Comparison of tumor characteristics between patients with ycN0ypN0 and ycN0ypN+.

Characteristics	No. of patients (%)		*p*
	ycN0ypN0 (n = 63)	ycN0ypN+ (n = 61)	
**Clinical T category at diagnosis**			
T1	7 (11.1)	6 (9.8)	0.31
T2	50 (79.3)	42 (68.9)	
T3	4 (6.3)	7 (11.5)	
T4	2 (3.2)	6 (9.8)	
**Tumor subtype**			
HR+/HER2−	22 (34.9)	52 (85.2)	< 0.001
HR+/HER2+	17 (27.0)	2 (3.3)	
HR−/HER2+	13 (20.6)	2 (3.3)	
HR−/HER2−	11 (17.5)	5 (8.2)	
**Tumor histology**			
IDC	57 (90.5)	52 (85.2)	0.39
ILC	2 (3.2)	1 (1.6)	
Others	4 (6.3)	8 (13.1)	
**No. of positive nodes before NAC as assessed by AUS**			
1	20 (31.7)	22 (36.0)	0.70
2	17 (27.0)	11 (18.0)	
3	7 (1.1)	8 (13.1)	
≧4	19 (30.2)	20 (32.8)	
**Tumor response as assessed by MRI**			
CR	20 (31.7)	3 (4.9)	0.0003
PR	42 (66.7)	50 (82.0)	
SD	1 (1.6)	7 (11.5)	
PD	0	1 (1.6)	

*HR* hormone receptor, *HER2* human epidermal growth factor receptor 2, *IDC* invasive ductal carcinoma, *ILC* invasive lobular carcinoma, *AUS* axillary ultrasound, *NAC* neoadjuvant chemotherapy, *ALND* axillary lymph node dissection, *CR* complete response, *PR* partial response, *SD* stable disease, *PD* progressive disease.

## Discussion

The use of SNB following NAC for patients with cN1 breast cancer is controversial because clinical trials have shown that the FNR exceeds the prespecified threshold of 10%^3–5^. To decrease FNR, dual-agent mapping, examining more than two sentinel lymph nodes (SLNs)^3–5,7^, targeted axillary dissection (TAD) placement including MARI, and tattooing procedure have been suggested^8–12^. When limited to patients with more than two SLNs removed, the FNR was 7.3% and 9.1% in the SENTINA and ACOSOG Z1071 trails, respectively^3,4^. However, in these two trials, these were unplanned analyses and only 56% and 34% of patients had more than two SLNs removed, respectively^13^. In addition, in SENTINA, lymph node status was not confirmed pathologically but suspected radiologically. Caudle et al. reported that the FNR decreased to as low as 2% using TAD^8^; however, TAD has limitations in that it requires extra procedures, such as clip placement at the time of biopsy and seed placement before surgery^14^. In addition, the response to NAC may differ among metastatic nodes in patients with multiple metastatic nodes^15^.

Another promising strategy to reduce FNR is preoperative axillary assessment by imaging. In the ACOSOG Z1071 trial, patients underwent SNB regardless of NAC response^4^. Following a secondary analysis of the trial, ACOSOG investigators reported that FNR was reduced to 9.8% by considering AUS assessment following NAC^6^. Similarly, in the SN FNAC trial, FNR was decreased to 2.7% by taking AUS assessment into account^16^. Although both studies showed that AUS reduced FNR, investigators took the position that US alone is not accurate enough to assess axillary response. Admittedly, the accurate prediction rate of node-negative status after NAC by AUS varies ranging from 46.2 to 89.6% in a number of studies^17–19^. Similarly, we found in our cohort that the NPV by AUS after NAC was 50.8%. However, it is conceivable that improvements of NPV by AUS would lead to decreased FNR of SNB following NAC. In this context, we demonstrated that the NPV by AUS after NAC was improved by addition of subtype information and breast tumor response to NAC by MRI. In fact, we found that the NPV was over 85% in HER2-positive cases in our cohort. These results suggest that the FNR of SNB after NAC can be reduced in patients with HER2-positive breast cancer under AUS determination of no lymph node metastasis after NAC. Moreover, we showed that all patients with HER2+ or HR−/HER2− disease and identified as ycN0 by AUS and cCR in primary tumor by MRI showed ypN0, indicating that these patients may be good candidates not requiring axillary surgery in the future.

Peppe et al. reported that there were notable variations in NPV and PPV by AUS according to tumor subtype, with HER2+ cases having a higher NPV, which is concordant with our results. However, other related factors were not investigated^19^. Several studies have reported factors associated with pathological response in nodes following NAC. Barrio et al. revealed that patients with HR+ breast cancer or those without breast pCR were less likely to have nodal pCR (*p* = 0.05); there was no evidence for other associative factors^20^. Tadros et al. reported that patients without breast pCR had a relative risk of 7.4 (95% confidence interval, 3.7–14.8; *p* < 0.001) to remaining positive for nodal metastases compared with those with breast pCR^2^. These results support our findings that accurate prediction of nodal pCR by AUS depends on subtype and breast tumor response as determined by MRI.

There are several limitations of our study. First, our sample size was modest. Particularly, in the subgroup analysis with subtypes and tumor response, the sample size in each subgroup was small. Thus, this study cannot make a solid conclusion. Because it is important to determine patients suitable for SNB after NAC, a larger study is necessary to ensure a sufficient sample size for subgroup analysis considering subtypes and breast tumor response. Second, this was a single institutional study so caution is warranted when considering the results in other institutions. However, it is worth noting that imaging diagnosis, such as AUS and breast MRI, was determined by consistent criteria in the single institution.

In conclusion, we found that the accurate prediction rate of node-negative status by AUS after NAC was subtype-dependent and highest in patients with HER2+ breast cancer. We also found that this increased by combining US assessment with breast tumor response determined by MRI. Regarding FNR reduction post-NAC, it will be of clinical value to take tumor subtype and primary tumor response using MRI into account to identify patients for SNB after NAC. In the future, it would be possible that patients with HER2+ or HR−/HER2− breast cancer who achieve ycN0 by AUS and cCR in primary tumor by MRI may be spared from axillary surgery. However, to better clarify optimal axillary management for patients with clinically node-positive breast cancer that converts to node negative after NAC, further prospective data considering clinical stage, molecular subtype, and clinical response in both breast and LNs is needed.
